# Association between fetal-type posterior cerebral artery variants and NIHSS-defined stroke severity in acute posterior circulation infarction

**DOI:** 10.3389/fneur.2026.1728595

**Published:** 2026-09-01

**Authors:** Jun He, Tao Hao, Wenjuan Li, Yu Liu, Yongandai Liu, Xiaoli Zhou

**Affiliations:** 1 Department of Neurology, Hubei Provincial Hospital of Traditional Chinese Medicine, Wuhan, Hubei, China; 2 Department of Neurology, Baoji High-tech Hospital, Baoji, Shaanxi, China; 3 Department of Radiology, Hubei Provincial Hospital of Traditional Chinese Medicine, Wuhan, Hubei, China; 4 College of Traditional Chinese Medicine, Hubei University of Chinese Medicine, Wuhan, Hubei, China; 5 Hubei Shizhen Laboratory, Hubei University of Chinese Medicine, Affiliated Hospital of Hubei University of Traditional Chinese Medicine (Hubei Provincial Hospital of Traditional Chinese Medicine), Wuhan, Hubei, China

**Keywords:** associated factors, connect, fetal-type posterior cerebral artery, posterior circulation infarction, stroke severity

## Abstract

**Objective:**

To investigate factors associated with stroke severity among patients with acute posterior circulation infarction (APCI) and to evaluate the association between fetal-type posterior cerebral artery (FTP) variants and NIHSS-defined stroke severity.

**Methods:**

A retrospective cohort of patients diagnosed with APCI by cranial magnetic resonance imaging (MRI) was selected from the two participating hospitals. The cohort included 161 patients (114 men and 47 women; mean age, 68.12 ± 15.81 years), with a mean admission NIHSS score of 6.28 ± 2.50. Based on magnetic resonance angiography findings, patients were categorized into an FTP group and a non-FTP group. Demographic characteristics, clinical data, and stroke severity as assessed by the National Institutes of Health Stroke Scale (NIHSS) were compared between the two groups. Logistic regression analysis was used to identify factors associated with moderate-to-severe stroke and to evaluate the association between FTP and NIHSS-defined stroke severity.

**Results:**

Univariate analysis revealed significant differences between the mild and moderate-to-severe groups in fasting blood glucose (5.71 ± 2.06 vs. 7.15 ± 1.74 mmol/L, *p* = 0.021), uric acid (284.24 ± 85.38 vs. 345.87 ± 86.27 μmol/L, *p* = 0.019), and prevalence of FTP (*p* < 0.001). Multivariable analysis showed that FTP was independently associated with moderate-to-severe stroke (adjusted OR = 5.604, 95% CI: 2.519–12.465, *p* < 0.001). There was a statistically significant difference in NIHSS scores between FTP and non-FTP patients (FTP: 8.55 ± 2.06 vs. non-FTP: 5.14 ± 1.84, *p* < 0.001).

**Conclusion:**

Among patients with APCI, FTP was independently associated with greater admission NIHSS-defined stroke severity. FTP may be a potential imaging marker of severity, but the limitations of admission NIHSS in posterior-circulation stroke, unavailable quantitative imaging covariates, and the retrospective design require cautious interpretation and prospective multicenter validation.

## 1 Introduction

According to the Global Burden of Disease Study 2016, China was estimated to have the highest lifetime risk of stroke worldwide (1). Data from the national mortality surveillance system in 2018 indicated that cerebrovascular disease (stroke) ranked as the third leading cause of death among Chinese residents, with its mortality rate increasing with age (2). Estimates from the Global Burden of Disease Study 2019 reported a stroke incidence rate of 276.7/100,000 and a mortality rate of 153.9/100,000 in China for the year 2019 (3), highlighting the substantial challenges currently faced in stroke prevention and management in China. Approximately 70% of strokes in China are acute ischemic strokes (4). Ischemic stroke results from the occlusion of a cerebral artery by thrombosis or embolism, leading to the interruption of cerebral blood flow. This causes ischemic and hypoxic damage to brain tissue, subsequently triggering neuropathological changes such as cerebral edema, neuroinflammation, and neuronal cell death, ultimately leading to severe neurological deficits (5). Ischemic stroke occurring in the territories supplied by the vertebrobasilar artery and the posterior cerebral artery is collectively termed posterior circulation ischemic stroke, accounting for approximately 20–30% of all ischemic strokes (6). It is classified based on symptom duration into posterior circulation transient ischemic attack (TIA) and acute posterior circulation infarction (APCI). Compared with the anterior circulation, the posterior circulation has distinct anatomical and clinical characteristics, and posterior circulation ischemic stroke may present with a wide spectrum of symptoms, contributing to diagnostic challenges and potential delays in recognition (7–9). The clinical presentation of posterior circulation ischemic stroke is often diverse and non-specific, contributing to high rates of underdiagnosis and misdiagnosis during early assessment, which can delay treatment. Furthermore, due to the complex anatomy of the posterior circulation—particularly the dense concentration of motor and sensory nerve fibers in the brainstem—posterior circulation ischemic stroke may be associated with poorer functional outcomes compared to anterior circulation ischemic stroke (10).

In recent years, the relationship between congenital vascular variants and acute ischemic stroke has garnered significant attention. Studies have demonstrated a close association between variants of the posterior cerebral artery and the occurrence of acute ischemic stroke (11, 12). The fetal-type posterior cerebral artery (FTP) represents the most frequent variant within the carotid artery system (13). It is defined as an anomalous posterior cerebral artery characterized by the persistent patency of the post-communicating segment originating from the embryonic period, concomitant with either absence or hypoplasia of the P1 segment. In the literature, FTP is typically differentiated into complete FTP (cFTP) and partial FTP (pFTP) based on the status of the pre-communicating (P1) segment. In cFTP, the P1 segment is absent, with no connection to the basilar artery, and the vessel originates entirely from the internal carotid artery system. In pFTP, although present, the P1 segment is hypoplastic; consequently, the blood supply is not derived exclusively from the internal carotid artery system and remains influenced by the basilar artery. Furthermore, when FTP is present unilaterally, it is termed unilateral FTP, whereas bilateral presence is referred to as bilateral FTP.

Current studies, both domestic and international, have reported on the impact of FTP on acute ischemic stroke, yet their conclusions remain inconsistent. Previous research has predominantly focused on acute anterior circulation infarction, with comparatively less attention devoted to acute posterior circulation infarction. Unlike the former, acute posterior circulation infarction is characterized by sudden onset and non-specific symptoms, often leading to difficulties in early recognition and accurate management, which significantly increases patient mortality and disability rates, thereby imposing a substantial healthcare burden on families and society. Consequently, evaluating the association between FTP and stroke severity among patients with acute posterior circulation infarction is clinically relevant. Based on this rationale, this study retrospectively collected clinical data from patients with acute posterior circulation infarction to analyze the correlation between the presence of FTP and stroke severity, with the aim of informing the clinical assessment of stroke severity in this population.

## 2 Materials and methods

### 2.1 Study subjects

Using a convenience sampling method within a retrospective study design, patients who underwent magnetic resonance imaging in the Department of Neurology at both Hubei Provincial Hospital of Traditional Chinese Medicine and Baoji High-tech Hospital between January 1, 2023, and December 31, 2024, were selected. Inclusion criteria: (1) met the diagnostic criteria for cerebral infarction established by the 4th National Cerebrovascular Disease Conference (14) and exhibited sensory-motor or cerebellar dysfunction, with the infarct located in the posterior-circulation territory as confirmed by MRI and diffusion-weighted imaging (DWI); (2) presented with focal neurological deficits; (3) had complete clinical and imaging information; and (4) voluntarily signed an informed-consent form. Exclusion criteria: (1) with other cerebrovascular variations or cardiovascular-related diseases; (2) patients with occlusion or severe stenosis of the internal carotid artery (ICA) and/or anterior cerebral artery (ACA), middle cerebral artery (MCA), vertebrobasilar artery (VBA); (3) who received thrombolytic and/or interventional therapy during the acute cerebral infarction phase; (4) complicated with anterior circulation cerebral infarction; (5) patients with severe organ failure, malignant tumors, autoimmune diseases, etc. To minimize confounding from major large-artery steno-occlusive disease and potential tandem lesions—which may strongly influence PCI severity independent of congenital vascular variants—patients with occlusion or severe stenosis of major intracranial arteries, including the vertebrobasilar system, were excluded. This study was approved by the Hospital Ethics Committee.

### 2.2 Study method

Magnetic resonance imaging was performed using a German-manufactured Philips Achieva 3.0 T superconducting MRI scanner equipped with a head surface coil and a phased-array coil. Axial spin-echo sequences were applied as follows: T1-weighted imaging (T1W) with repetition time (TR) = 530 ms and echo time (TE) = 7.2 ms; T2-weighted imaging (T2WI) with TR = 5,200 ms, TE = 120 ms, slice thickness = 4 mm, inter-slice gap = 2 mm, matrix = 512 × 512; diffusion-weighted imaging (DWI) with TR = 4,200 ms, TE = 63 ms, slice thickness = 5 mm, inter-slice gap = 3 mm, matrix = 256 × 256, field of view (FOV) = 23 cm × 23 cm, b-value = 1,000 s/mm^2^. Routine imaging planes included axial and transverse sections, with a subset of examinations also acquiring coronal sections; for these, slice thickness was 4 mm with a gap ranging from 0 to 3 mm. Magnetic resonance angiography (MRA) employed three-dimensional time-of-flight (3D-TOF) acquisition (TR = 17.24 ms, TE = 6.23 ms, FOV = 260 mm × 260 mm, slice thickness = 1.9 mm, inter-slice gap = 0.95 mm, matrix = 256 × 256). Based on MRA findings, participants were classified into an FTP group and a non-FTP group. All imaging data were independently interpreted by two experienced neurologists at different times and locations; in cases of discordance, a third senior physician performed a consensus reading.

### 2.3 Data collection

Patient information was retrospectively collected, including age, sex, history of hypertension, hyperlipidemia, diabetes mellitus, smoking, blood pressure, lipid profile, blood glucose, and stroke severity. Admission neurological deficit severity was assessed using the National Institutes of Health Stroke Scale (NIHSS) (15), encompassing 11 domains: level of consciousness, gaze, visual fields, facial palsy, upper-extremity motor function, lower-extremity motor function, limb ataxia, sensory function, language, dysarthria, and neglect. Scores < 8 were classified as mild stroke, whereas scores ≥ 8 indicated moderate-to-severe stroke. The primary infarct site was extracted from the MRI-DWI report for all participants. For descriptive regional comparison, primary infarct sites were grouped into three anatomically defined regions: brainstem, including the medulla, pons, and midbrain; cerebellar, including the posterior inferior and superior cerebellum; and supratentorial posterior-circulation regions, including the thalamus, occipital lobe, and temporal lobe. Available case-level MRI-DWI report wording was additionally compiled in Supplementary Table S1 for transparency. For an additional exploratory stratified analysis, 75 participants from one participating center with linkable case-level NIHSS scores and MRI-DWI lesion descriptions were categorized as having cerebellar, brainstem, occipital-lobe, or multi-site lesions according to the documented imaging wording.

### 2.4 Statistical analysis

Data analysis was performed using SPSS 26.0. Normality was assessed using the Kolmogorov–Smirnov test. Continuous variables were summarized as mean ± standard deviation (SD) and compared using the independent-samples *t*-test when distributional assumptions were met. Categorical variables were presented as counts and percentages and compared using the *χ*^2^ test; Fisher’s exact test was used when expected cell counts were <5. Stroke severity was dichotomized as mild (NIHSS < 8) versus moderate-to-severe (NIHSS ≥ 8). Multivariable logistic regression was performed with moderate-to-severe stroke as the dependent variable. The model included FTP status and clinical covariates selected on the basis of clinical relevance and established vascular risk factors rather than automatic univariate screening: age, sex, hypertension, diabetes mellitus, smoking history, and prior stroke history. Although fasting blood glucose and uric acid differed significantly between severity groups in univariate comparisons, they were retained as exploratory findings and were not included in the multivariable model, which was kept parsimonious in view of the 69 moderate-to-severe events. Primary infarct-site and grouped regional distributions were compared descriptively between FTP and non-FTP groups. Infarct location was not added to the multivariable model because the recorded anatomical categories were heterogeneous, multifocal lesions were common in the available descriptions, and additional location parameters could reduce model stability in this event-limited cohort. In the subset of 75 participants with linkable case-level NIHSS scores and lesion-location descriptions, the distribution of mild versus moderate-to-severe stroke across documented lesion-location categories was compared using the Fisher–Freeman–Halton exact test because several cells contained small numbers. This stratified analysis was considered exploratory and was not used to replace the complete-cohort multivariable model. Adjusted odds ratios (ORs) with 95% confidence intervals (CIs) were reported. Model fit was assessed using the Hosmer–Lemeshow goodness-of-fit test. A two-sided *p* < 0.05 was considered statistically significant.

## 3 Results

### 3.1 Comparison of clinical data among patients with different stroke severity

The results showed that the mild group comprised 66 males and 26 females, with a mean age of 67.38 ± 15.41 years; the moderate to severe group comprised 48 males and 21 females, with a mean age of 69.11 ± 16.39 years. Significant inter-group differences were observed in fasting blood glucose (5.71 ± 2.06 vs. 7.15 ± 1.74 mmol/L, *p* = 0.021), uric acid (284.24 ± 85.38 vs. 345.87 ± 86.27 μmol/L, *p* = 0.019), smoking history (*p* = 0.011), and FTP ratio (*p* < 0.001) (see Table 1). Available case-level MRI-DWI report wording was compiled for 143 of the 161 participants and is provided in Supplementary Table S1 for transparency.

**Table 1:** Comparison of clinical data of patients with different degrees of stroke.

Item	Mild severity group (*n* = 92)	Moderate to severe group (*n* = 69)	*χ^2^/t*	*P-*value
Gender (male/female)	66/26	48/21	0.090	0.764
Age (year)	67.38 ± 15.41	69.11 ± 16.39	0.741	0.364
Hypertension	68	46	1.002	0.317
Diabetes mellitus	26	21	0.090	0.764
Coronary heart disease	21	16	0.003	0.957
History of stroke	45	24	3.215	0.073
BMI (kg/m^2^)	23.52 ± 2.89	23.05 ± 3.07	1.041	0.250
History of smoke	39	16	6.464	0.011
History of drink	18	14	0.013	0.909
FTP	17	37	21.848	<0.001
Systolic pressure (mmHg)	148.41 ± 20.30	147.39 ± 23.07	0.578	0.589
Diastolic pressure (mmHg)	83.53 ± 10.10	82.79 ± 9.06	0.307	0.579
Fasting blood glucose (mmol/L)	5.71 ± 2.06	7.15 ± 1.74	5.727	0.021
Low density lipoprotein (mmol/L)	2.80 ± 0.94	2.87 ± 0.88	1.291	0.262
High density lipoprotein (mmol/L)	1.13 ± 0.30	1.12 ± 0.30	0.074	0.841
Uric acid (μmol/L)	284.24 ± 85.38	345.87 ± 86.27	4.972	0.019
Urea (mmol/L)	5.91 ± 3.22	5.67 ± 2.09	0.971	0.325
Creatinine (μmol/L)	75.70 ± 29.43	70.68 ± 21.64	1.175	0.197
Fibrin (g/L)	3.27 ± 0.72	2.99 ± 0.83	1.089	0.392
Homocysteine (μmol/L)	17.40 ± 11.60	16.74 ± 7.55	0.427	0.566

Continuous variables are presented as mean ± SD and were compared using the independent-samples *t*-test after assessment of normality (Kolmogorov–Smirnov test). Categorical variables are presented as n and were compared using the *χ*^2^ test; Fisher’s exact test was used when expected cell counts were <5. Two-sided *p* < 0.05 was considered statistically significant.

### 3.2 Comparison of FTP subgroup data

Among the 54 patients with FTP, 34 were classified as pFTP and 20 as cFTP. The NIHSS score was significantly lower in the pFTP subgroup than in the cFTP subgroup (7.53 ± 0.71 vs. 8.85 ± 2.66, *p* = 0.008). However, no significant difference was observed in stroke severity distribution between the two subgroups (*p* = 0.181) (see Table 2).

**Table 2:** Comparison of FTP subgroup data.

Project	pFTP (*n* = 34)	cFTP (*n* = 20)	*χ^2^/t*	*P-*value
NIHSS Score	7.53 ± 0.71	8.85 ± 2.66	−2.750	0.008
Stroke Severity			1.787	0.181
Mild	20	8		
Moderate to Severe	14	12		

Continuous variables (NIHSS score) are expressed as mean ± standard deviation (SD) and were compared using the independent-samples *t*-test after normality assessment. Categorical variables (stroke severity) are expressed as counts (n) and were analyzed using the *χ*^2^ test. Two-sided *P* < 0.05 was considered statistically significant.

### 3.3 Multivariable logistic regression analysis of factors associated with stroke severity

Using moderate-to-severe stroke as the dependent variable, the multivariable logistic regression model showed that FTP was associated with higher odds of moderate-to-severe stroke after adjustment for age, sex, hypertension, diabetes mellitus, smoking history, and prior stroke history (adjusted OR = 5.604, 95% CI: 2.519–12.465; *p* < 0.001). The model showed acceptable fit according to the Hosmer–Lemeshow test (*χ*^2^ = 7.258, df = 8, *p* = 0.509, Table 3).

**Table 3:** Multivariable logistic regression analysis of factors associated with moderate-to-severe APCI.

Variable	*B*	S. E.	Wald	*P*-value	OR	95% CI
FTP	1.723	0.408	17.853	<0.001	5.604	2.519–12.465
Age	−0.011	0.016	0.498	0.480	0.989	0.959–1.020
Sex, male vs. female	−0.245	0.488	0.252	0.616	0.783	0.301–2.037
Hypertension	−0.061	0.417	0.021	0.884	0.941	0.415–2.133
Diabetes mellitus	−0.311	0.437	0.506	0.477	0.733	0.311–1.726
Smoking history	−0.831	0.438	3.602	0.058	0.436	0.185–1.028
History of stroke	−0.323	0.400	0.652	0.420	0.724	0.331–1.586

Multivariable logistic regression was performed with moderate-to-severe stroke, defined as NIHSS ≥ 8, as the dependent variable. Covariates included FTP status, age, sex, hypertension, diabetes mellitus, smoking history, and prior stroke history. Covariates were selected on the basis of clinical relevance and established vascular risk factors rather than automatic univariate screening. Fasting blood glucose and uric acid were retained as exploratory univariate findings and were not included in the model, which was kept parsimonious in view of the 69 moderate-to-severe events. Results are presented as regression coefficient (B), standard error (S. E.), Wald statistic, odds ratio (OR), and 95% confidence interval (CI). The Hosmer–Lemeshow goodness-of-fit test yielded *χ*^2^ = 7.258, df = 8, and *p* = 0.509. A two-sided *p* < 0.05 was considered statistically significant.

### 3.4 Comparison of data between FTP and non-FTP patients

A total of 54 patients with FTP and 107 patients without FTP were enrolled. The FTP group comprised 33 males and 21 females with a mean age of 69.33 ± 12.73 years, whereas the non-FTP group comprised 81 males and 26 females with a mean age of 68.37 ± 12.16 years. NIHSS scores were higher in the FTP group than in the non-FTP group (8.55 ± 2.06 vs. 5.14 ± 1.84, *p* < 0.001). The distribution of the eight primary infarct sites did not differ significantly between the groups (*p* = 0.277). After these sites were combined into brainstem, cerebellar, and supratentorial posterior-circulation regions for descriptive comparison, the regional distribution also did not differ between the FTP and non-FTP groups (*p* = 0.824). No significant inter-group differences were found for the other clinical characteristics presented in Table 4.

**Table 4:** Comparison of clinical and primary infarct-location characteristics between FTP and non-FTP groups.

Item	FTP group (*n* = 54)	Non-FTP group (*n* = 107)	*χ*^2^/*t*	*P*-value
Sex, male/female	33/21	81/26	3.696	0.055
Age, years	69.33 ± 12.73	68.37 ± 12.16	0.465	0.642
Hypertension	37	77	0.506	0.650
Diabetes mellitus	17	30	0.506	0.650
Coronary heart disease	15	22	1.056	0.304
History of stroke	28	41	2.684	0.101
BMI, kg/m^2^	22.36 ± 3.28	23.17 ± 3.16	1.518	0.131
Smoking history	15	40	1.472	0.225
Drinking history	12	20	0.281	0.596
Systolic blood pressure, mmHg	144.30 ± 22.74	147.44 ± 20.69	0.880	0.380
Diastolic blood pressure, mmHg	82.70 ± 13.23	87.13 ± 13.99	1.930	0.055
Fasting blood glucose, mmol/L	6.09 ± 2.31	6.55 ± 2.76	1.048	0.296
Low-density lipoprotein, mmol/L	2.93 ± 1.05	2.83 ± 1.04	0.605	0.546
High-density lipoprotein, mmol/L	1.15 ± 0.35	1.06 ± 0.28	1.753	0.082
Uric acid, μmol/L	311.55 ± 89.15	327.90 ± 96.43	1.035	0.302
Urea, mmol/L	5.82 ± 4.69	5.81 ± 2.00	0.016	0.988
Creatinine, μmol/L	70.01 ± 23.09	78.48 ± 39.15	1.465	0.145
Fibrin, g/L	3.18 ± 0.85	3.26 ± 1.00	0.464	0.643
Homocysteine, μmol/L	15.32 ± 7.21	19.62 ± 16.32	1.844	0.067
NIHSS score	8.55 ± 2.06	5.14 ± 1.84	9.717	<0.001
Primary infarct site			—	0.277
Medulla	2	12		
Posterior inferior cerebellum	11	16		
Pons	14	27		
Thalamus	1	6		
Occipital lobe	4	13		
Temporal lobe	13	13		
Midbrain	5	8		
Superior cerebellum	4	12		
Grouped primary infarct region			0.386	0.824
Brainstem	21	47		
Cerebellar	15	28		
Supratentorial posterior circulation	18	32		

Continuous variables are presented as mean ± SD and were compared using the independent-samples *t*-test after assessment of normality using the Kolmogorov–Smirnov test. Categorical variables are presented as n and were compared using the *χ*^2^ test; Fisher’s exact test was used when expected cell counts were <5. Brainstem infarctions included lesions in the medulla, pons, and midbrain; cerebellar infarctions included lesions in the posterior inferior and superior cerebellum; and supratentorial posterior-circulation infarctions included lesions in the thalamus, occipital lobe, and temporal lobe. The eight-category primary infarct-site distribution was retained to show the original anatomical data, and the three-category regional variable was used for descriptive comparison. Supplementary Table S1 provides available case-level MRI-DWI report wording for 143 participants and is presented solely for transparency. Two-sided *P* < 0.05 was considered statistically significant.

### 3.5 Exploratory stratified analysis of stroke severity by lesion-location category

Among the 75 participants with linkable case-level NIHSS scores and MRI-DWI lesion descriptions, moderate-to-severe stroke occurred in 16 of 23 patients (69.6%) with multi-site lesions, 1 of 3 patients (33.3%) with brainstem lesions, 1 of 48 patients (2.1%) with cerebellar lesions, and 0 of 1 patient with an occipital-lobe lesion. The distribution of NIHSS-defined stroke severity differed across the documented lesion-location categories (Fisher–Freeman–Halton exact *p* < 0.001; Supplementary Table S2). Because this analysis was restricted to a one-center subset and several categories contained few patients, the findings were considered exploratory.

## 4 Discussion

The FTP is a common variant of the Circle of Willis, defined as a situation in which the entire or majority of the posterior cerebral artery’s blood supply originates from the ipsilateral internal carotid artery. This variant not only expands the perfusion territory of the internal carotid artery but also impedes the formation of leptomeningeal collaterals between the anterior and posterior circulations. When an FTP is present, hypoplasia or absence of the P1 segment prevents the thalamoperforating and posterior choroidal branches from establishing compensatory collateral pathways, leading to thalamic hypoperfusion and secondary thalamic infarction (16). Moreover, concomitant vertebral artery hypoplasia associated with FTP diminishes the capacity for collateral compensation, reduces local cerebral perfusion, and consequently produces neurological deficits related to the affected brain regions, underscoring a close link between FTP and cerebral infarction. Prior studies have also demonstrated that individuals with vertebral artery hypoplasia have a higher probability of posterior circulation ischemia, indicating an association between FTP and ischemic stroke (17). Within APCI cohorts, FTP has been associated with greater neurological deficit severity and may therefore be relevant to severity assessment, although it should not be interpreted as evidence of causation or of APCI occurrence.

Compared with acute anterior circulation cerebral infarction, APCI generally presents with a more severe clinical course. Investigating factors associated with its severity and prognosis is therefore of great importance, although existing studies have yielded conflicting conclusions (18, 19). Some investigators have reported that patients with FTP have significantly higher rates of smoking, alcohol consumption, hypertension, diabetes mellitus, and hyperhomocysteinemia than non-FTP patients (20, 21). Conversely, other studies have found no statistically significant differences in baseline clinical characteristics between FTP and non-FTP groups (22, 23). In the present study, the baseline clinical characteristics were largely comparable between the FTP and non-FTP groups. Specifically, no statistically significant inter-group differences were observed in sex, age, hypertension, diabetes mellitus, coronary heart disease, history of stroke, BMI, smoking history, drinking history, blood pressure, fasting blood glucose, lipid parameters, uric acid, urea, creatinine, fibrin, homocysteine, or infarct site distribution (all *p* > 0.05). The only significant difference between the two groups was the NIHSS score, which was higher in the FTP group than in the non-FTP group. These findings suggest that FTP may be more closely related to stroke severity than to differences in baseline vascular risk profiles within this APCI cohort.

In this study, stroke severity was assessed using the NIHSS. The moderate-to-severe stroke group had higher fasting blood glucose levels, uric acid concentrations, and prevalence of FTP in the univariate comparisons. These laboratory variables were treated as exploratory univariate findings rather than interpreted as independent factors. The multivariable model was constructed using clinically relevant vascular covariates rather than automatic selection based on univariate *p*-values and was kept parsimonious given the 69 moderate-to-severe events. In the multivariable model incorporating age, sex, hypertension, diabetes mellitus, smoking history, and prior stroke history, FTP was associated with moderate-to-severe stroke (adjusted OR = 5.604, 95% CI: 2.519–12.465; *p* < 0.001). Because infarct location may materially influence NIHSS scores, primary infarct-site and grouped regional distributions were examined descriptively. Neither distribution differed significantly between the FTP and non-FTP groups. In the exploratory stratified analysis of the 75-case subset, moderate-to-severe stroke was concentrated among patients with multi-site lesion descriptions, whereas it was uncommon among patients with isolated cerebellar lesions. This finding supports the clinical relevance of lesion distribution to NIHSS-defined severity. However, the subset analysis was restricted to one participating center, several lesion categories were sparse, and standardized data on infarct volume and the involvement of specific strategic structures were unavailable. However, infarct location was not entered into the multivariable model because of heterogeneous anatomical categories, frequent multifocal lesions in the available descriptions, and the limited number of outcome events. Residual anatomical and metabolic confounding therefore cannot be excluded. NIHSS-based dichotomization may also incompletely represent the phenotypic heterogeneity of posterior circulation infarction. Therefore, the findings should be interpreted as an association with NIHSS-defined severity rather than evidence of causality. The putative mechanisms by which FTP may influence neurological deficit severity include altered collateral pathways and hemodynamic redistribution between the anterior and posterior circulations (24, 25), as well as the frequent coexistence of vertebrobasilar hypoplasia and reduced posterior-circulation perfusion reserve (26, 27). These mechanisms may contribute to variation in neurological deficit severity among patients with APCI, but require confirmation in studies incorporating quantitative lesion characteristics and cerebral perfusion measurements. Admission NIHSS may underrepresent posterior-circulation manifestations such as gait or truncal ataxia, dysphagia, diplopia, vertigo, and cranial nerve deficits; therefore, NIHSS-defined severity does not capture the full clinical burden of APCI (7–10). Accordingly, FTP should currently be regarded as a hypothesis-generating imaging correlate rather than a stand-alone marker for clinical decision-making.

Existing studies have investigated the association between the FTP and ischemic stroke (28). Some investigators have reported that patients with FTP have an increased risk of acute cerebral infarction (29) and are more prone to watershed infarcts (30). Hoque et al. (31) noted a dual effect of FTP on stroke: when atherosclerosis is present in the posterior circulation, the ipsilateral ICA supply via FTP may reduce the risk of occipital infarction caused by emboli dislodging from the posterior circulation (32); however, if FTP coexists with ipsilateral internal carotid artery stenosis or occlusion, it may precipitate watershed infarction in the PCA supplied territory. Roshan et al. also found that the pFTP is associated with a higher incidence of ischemic stroke, whereas the cFTP shows no clear relationship (33). In the present study, subgroup analysis within the FTP cohort showed that the NIHSS score was significantly lower in patients with pFTP than in those with cFTP, whereas the distribution of stroke severity did not differ significantly between the two subgroups. This finding suggests that although FTP subtype may be associated with differences in neurological deficit burden, the relationship between subtype and categorized stroke severity remains uncertain and requires further validation in larger cohorts.

This study has several limitations. (1) The retrospective two-center design and requirement for complete clinical and imaging records may have introduced selection bias and may limit the representativeness of the study population. Nevertheless, inclusion of patients from two independent hospitals improves the clinical relevance of the findings, which should be further examined in larger multicenter cohorts. (2) Patients who received thrombolysis or endovascular treatment and those with major vertebrobasilar steno-occlusive disease were excluded to reduce treatment-related and large-vessel confounding and to evaluate the association between FTP and neurological deficit severity in a relatively homogeneous non-reperfused APCI population. Accordingly, the generalizability of the findings to reperfusion-treated patients and those with major posterior-circulation large-artery disease remains to be established. (3) The overall sample size was modest, and the numbers of patients in some FTP subtype and lesion-location categories were limited. The relatively wide confidence interval therefore indicates that the magnitude of the observed association requires confirmation, although the association remained statistically significant after adjustment for major clinical covariates. FTP subtype analyses should likewise be considered exploratory until validated in adequately powered cohorts. (4) Infarct-location distributions were examined descriptively in the complete cohort, and an additional exploratory subset analysis was performed in participants with linkable NIHSS and lesion-description data. However, standardized quantitative information on infarct volume, multifocality, bilateral involvement, strategic-structure involvement, collateral circulation, residual vascular occlusion, total lesion burden, and perfusion characteristics was not available across the entire cohort. Consequently, some residual anatomical confounding may remain. Fasting blood glucose and uric acid were also not included in the multivariable model, and their potential contribution should be evaluated in larger datasets. Future studies incorporating standardized lesion segmentation and prespecified location-, volume-, and perfusion-adjusted analyses will help refine the clinical interpretation of the association. (5) Stroke severity was assessed using admission NIHSS, a standardized and widely used measure of neurological deficit severity. However, NIHSS may be less sensitive to certain posterior-circulation manifestations and does not directly capture long-term functional recovery. Future prospective studies should therefore combine NIHSS with posterior-circulation-sensitive neurological assessments, quantitative imaging biomarkers, and standardized functional outcomes. Despite these limitations, the observed association provides clinically meaningful evidence that FTP may contribute useful imaging information to the early assessment of APCI severity.

## 5 Conclusion

Among patients with APCI, FTP was associated with greater admission NIHSS-defined stroke severity in the multivariable model. These findings support FTP as a potentially informative imaging marker that may complement early neurological assessment and severity stratification in APCI. Nevertheless, its clinical relevance should be interpreted in conjunction with neurological examination and conventional imaging findings. The relatively wide confidence interval, the recognized limitations of NIHSS in posterior-circulation stroke, and the absence of standardized quantitative imaging and metabolic variables indicate the need for further prospective multicenter validation rather than diminishing the observed association.

## Data Availability

The original contributions presented in the study are included in the article/Supplementary material, further inquiries can be directed to the corresponding author.
