# Supplementary material for: Association between fetal-type posterior cerebral artery variants and NIHSS-defined stroke severity in acute posterior circulation infarction
Source: Front Neurol. 2026 Sep 1;17:1728595. doi: 10.3389/fneur.2026.1728595 (PMC13575510; doi:10.3389/fneur.2026.1728595)
Supplement: Supplementary file 1 [file Table_1.DOCX]

**Supplementary Table S1. Available case-level infarct-location documentation (MRI-DWI) for included APCI patients (n = 143)**

| **Patient ID**  **(de-identified)** | **DWI lesion location**  **(verbatim from record)** | **Classified location category** | **Notes** |
| --- | --- | --- | --- |
| 428374 | Acute infarction in the left brainstem | Brainstem | — |
| 422633 | Acute infarction in the right brainstem | Brainstem | — |
| 420977 | Acute infarction in the brainstem | Brainstem | — |
| 418981 | Acute infarction in the brainstem | Brainstem | — |
| 416944 | Late subacute infarction in the right brainstem | Brainstem | Subacute stage (late) |
| 416718 | Acute infarction in the brainstem | Brainstem | — |
| 479103 | Pontine infarction (acute–subacute) | Brainstem | Pons (brainstem) |
| 345274 | Acute infarction in the brainstem | Brainstem | — |
| 344137 | Acute infarction in the right brainstem | Brainstem | — |
| 350130 | Pontine lacunar infarction (acute–subacute stage) | Brainstem | Lacunar infarction |
| 325710 | Late subacute lacunar infarction in the brainstem | Brainstem | Lacunar infarction; subacute stage (late) |
| 322321 | Acute lacunar infarction in the brainstem | Brainstem | Lacunar infarction |
| 320231 | Acute infarction in the brainstem | Brainstem | — |
| 311858 | Acute infarction in the pons | Brainstem | Pons (brainstem) |
| 327275 | Brainstem infarction (acute/subacute) | Brainstem | Mixed acute/subacute wording |
| 306089 | Acute infarction in the right brainstem | Brainstem | — |
| 350893 | Subacute infarction in the brainstem | Brainstem | Subacute stage |
| 343743 | Acute lacunar infarction in the brainstem | Brainstem | Lacunar infarction |
| 363629 | Acute or early subacute lacunar infarction in the left thalamus | Thalamus | Lacunar infarction; mixed acute/early subacute wording |
| 328099 | Acute infarction in the right thalamus | Thalamus | — |
| 321205 | Subacute infarction in the left thalamus | Thalamus | Subacute stage |
| 320270 | Acute lacunar infarction in the left thalamus | Thalamus | Lacunar infarction |
| 338493 | Focal fresh infarction in the right thalamus | Thalamus | Fresh infarct |
| 328982 | Infarction in the right thalamic region (acute–subacute stage) | Thalamus | — |
| 298931 | Acute infarction in the left thalamus | Thalamus | — |
| 295185 | Acute lacunar infarction in the left thalamus | Thalamus | Lacunar infarction |
| 289040 | Subacute infarction in the left thalamus | Thalamus | Subacute stage |
| 288394 | Acute infarction in the right thalamus | Thalamus | — |
| 424944 | Acute infarction in the left thalamus | Thalamus | — |
| 419784 | Acute infarction in the left thalamus | Thalamus | — |
| 417404 | Subacute lacunar infarct focus in the left thalamus | Thalamus | Lacunar infarction |
| 106815 | Thalamic lacunar infarction in the right thalamus (acute–subacute stage) | Thalamus | Lacunar infarction |
| 105672 | Early subacute lacunar infarction in the right thalamus (acute to early subacute stage) | Thalamus | Lacunar infarction |
| 103744 | Subacute infarction in the left thalamus | Thalamus | Subacute stage |
| 429452 | Infarction in the right thalamus (early subacute stage) | Thalamus | Subacute stage (early) |
| 403814 | Focal fresh infarction in the left thalamus | Thalamus | Fresh infarct |
| 394269 | Focal fresh infarction in the left thalamus | Thalamus | Fresh infarct |
| 355897 | Acute lacunar infarct focus in the right thalamus | Thalamus | Lacunar infarction |
| 380807 | Subacute infarction in the right thalamus | Thalamus | Subacute stage |
| 309205 | Large subacute infarction involving the left cerebellar hemisphere, cerebellar vermis, and middle cerebellar peduncle | Cerebellum | Subacute stage; multiple cerebellar structures |
| 297284 | Acute infarction in the right cerebellum | Cerebellum | — |
| 296667 | Acute lacunar infarction in the left cerebellum | Cerebellum | Lacunar infarction |
| 306857 | Suspected focal infarction in the right cerebellar hemisphere | Cerebellum | “Suspected” per record |
| 293487 | Acute infarction in the left cerebellar hemisphere | Cerebellum | — |
| 289351 | Subacute infarction in the right cerebellum | Cerebellum | Subacute stage |
| 288328 | Subacute infarction in the left cerebellum | Cerebellum | Subacute stage |
| 405348 | Cerebellar infarction (acute/subacute) in the left cerebellum | Cerebellum | Mixed acute/subacute wording |
| 497031 | Acute infarction in the cerebellum | Cerebellum | Laterality not specified |
| 115225 | Focal fresh infarction in the right cerebellar hemisphere | Cerebellum | Fresh infarct |
| 360959 | Fresh infarction in the left cerebellar hemisphere and left cerebellar vermis | Cerebellum | Multiple cerebellar structures |
| 356507 | Acute-stage infarction in the left cerebellum | Cerebellum | Acute-stage wording |
| 351820 | Acute-stage or early subacute infarction in the left cerebellum | Cerebellum | Mixed acute/early subacute wording |
| 406235 | Acute infarction in the right cerebellum | Cerebellum | — |
| 312093 | Acute lacunar infarction in the left cerebellar hemisphere | Cerebellum | Lacunar infarction |
| 355863 | Acute infarction in the left cerebellum | Cerebellum | — |
| 109367 | Multiple infarctions involving the cerebellar vermis, cerebellar tonsils, and cerebellar hemispheres (acute/subacute) | Cerebellum | Multiple cerebellar structures |
| 412794 | Large cerebellar infarction in the right cerebellum (acute–subacute stage) | Cerebellum | Large infarct |
| 406735 | Infarction in the left cerebellar hemisphere (partly acute–subacute, partly chronic) | Cerebellum | Mixed acute/subacute with chronic component |
| 368925 | Subacute infarction in the left parietal lobe | Anterior-circulation wording in original report | As documented in record |
| 303005 | Acute infarction in the right cerebellum and subacute infarction in the pons | Multi-site | Cerebellum + brainstem |
| 110179 | Multiple infarctions in the bilateral cerebellum and bilateral occipital lobes (acute–subacute stage) | Multi-site | — |
| 387693 | Acute infarction involving the left cerebellum, pons (brainstem), and right temporal lobe | Multi-site | — |
| 371996 | Multiple acute infarctions involving the bilateral cerebellum, right brainstem, bilateral occipital lobes, and thalamus | Multi-site | Multi-territory involvement per record |
| 404649 | Multiple lacunar infarctions at the right cerebellar margin (subacute–mid stage) with right parietal lobe changes | Multi-site | Subacute-mid stage wording; additional parietal change |
| 360480 | Subacute infarction involving the cerebellum and brainstem | Multi-site | — |
| 320287 | Acute infarction in the right cerebellum and multiple acute lacunar infarctions in the left fronto-temporo-parietal regions | Multi-site | Accompanying anterior-circulation wording in original report |
| 336604 | Infarction involving the right cerebellum and pons (acute/subacute stage) | Multi-site | Cerebellum + brainstem |
| 312063 | Infarctions involving the brainstem, right cerebral peduncle, and left occipital lobe (acute/subacute lacunar infarctions) | Multi-site | — |
| BJ001 | Acute large infarction involving the left cerebellar hemisphere, cerebellar vermis, cerebellar peduncle, pons, and bilateral thalamus. | Multi-site | Large lesion |
| BJ002 | Acute infarction involving the right cerebellum, and pons. | Multi-site | — |
| BJ003 | Acute infarction involving the right cerebellum. | Cerebellum | — |
| BJ004 | Acute lacunar infarction involving the left cerebellum. | Cerebellum | Lacunar infarction |
| BJ005 | Acute infarction involving the right pons, and right occipital lobe. | Multi-site | — |
| BJ006 | Acute infarction involving the left cerebellar hemisphere, and cerebellar peduncle. | Cerebellum | — |
| BJ007 | Subacute infarction involving the right midbrain, and pons. | Brainstem | Subacute-stage wording |
| BJ008 | Subacute infarction involving the left cerebellum. | Cerebellum | Subacute-stage wording |
| BJ009 | Acute infarction involving the left cerebellum, and pons. | Multi-site | — |
| BJ010 | Acute infarction involving the cerebellum. | Cerebellum | — |
| BJ011 | Fresh focal infarction involving the right cerebellar hemisphere. | Cerebellum | Fresh infarct wording |
| BJ012 | Fresh infarction involving the left cerebellar hemisphere, and left cerebellar vermis. | Cerebellum | Fresh infarct wording |
| BJ013 | Acute infarction involving the left cerebellum, right pons, and right midbrain. | Multi-site | — |
| BJ014 | Acute or early subacute infarction involving the left cerebellum. | Cerebellum | Subacute-stage wording |
| BJ015 | Acute infarction involving the right cerebellum. | Cerebellum | — |
| BJ016 | Acute lacunar infarction involving the left cerebellar hemisphere, pons, and right midbrain. | Multi-site | Lacunar infarction |
| BJ017 | Acute infarction involving the left cerebellum. | Cerebellum | — |
| BJ018 | Acute/subacute multiple infarction involving the cerebellar vermis, cerebellar tonsil, and cerebellar hemisphere. | Cerebellum | Multiple lesions; Subacute-stage wording |
| BJ019 | Acute large infarction involving the right cerebellum. | Cerebellum | Large lesion |
| BJ020 | Recorded infarction involving the left cerebellar hemisphere. | Cerebellum | — |
| BJ021 | Recorded infarction involving the left cerebellum. | Cerebellum | — |
| BJ022 | Acute infarction involving the right pons, right cerebellum, and right occipital lobe. | Multi-site | — |
| BJ023 | Acute infarction involving the right cerebellum. | Cerebellum | — |
| BJ024 | Acute infarction involving the right cerebellum. | Cerebellum | — |
| BJ025 | Acute lacunar infarction involving the left cerebellum, left pons, and left midbrain. | Multi-site | Lacunar infarction |
| BJ026 | Acute infarction involving the right occipital lobe. | Occipital lobe | — |
| BJ027 | Acute infarction involving the left cerebellar hemisphere. | Cerebellum | — |
| BJ028 | Acute infarction involving the right cerebellum. | Cerebellum | — |
| BJ029 | Acute infarction involving the left cerebellum. | Cerebellum | — |
| BJ030 | Acute infarction involving the left cerebellum. | Cerebellum | — |
| BJ031 | Acute infarction involving the right cerebellum, right pons, and midbrain. | Multi-site | — |
| BJ032 | Fresh focal infarction involving the right cerebellar hemisphere. | Cerebellum | Fresh infarct wording |
| BJ033 | Fresh infarction involving the left cerebellar hemisphere, and medulla. | Multi-site | Fresh infarct wording |
| BJ034 | Acute infarction involving the left cerebellum, and cerebellar peduncle. | Cerebellum | — |
| BJ035 | Acute infarction involving the left cerebellum, and left medulla. | Multi-site | — |
| BJ036 | Acute infarction involving the right cerebellum, and pons. | Multi-site | — |
| BJ037 | Acute lacunar infarction involving the left cerebellar hemisphere, and midbrain. | Multi-site | Lacunar infarction |
| BJ038 | Acute infarction involving the left cerebellum. | Cerebellum | — |
| BJ039 | Acute infarction involving the right cerebellar tonsil. | Cerebellum | — |
| BJ040 | Acute infarction involving the right cerebellum. | Cerebellum | — |
| BJ041 | Acute infarction involving the left cerebellar hemisphere. | Cerebellum | — |
| BJ042 | Acute infarction involving the right pons. | Brainstem | — |
| BJ043 | Acute infarction involving the left cerebellum. | Cerebellum | — |
| BJ044 | Acute infarction involving the left cerebellum. | Cerebellum | — |
| BJ045 | Acute infarction involving the right cerebellum. | Cerebellum | — |
| BJ046 | Fresh focal infarction involving the right cerebellar hemisphere. | Cerebellum | Fresh infarct wording |
| BJ047 | Fresh infarction involving the left cerebellar hemisphere. | Cerebellum | Fresh infarct wording |
| BJ048 | Acute infarction involving the left cerebellum, and cerebellar peduncle. | Cerebellum | — |
| BJ049 | Acute infarction involving the left cerebellum, and left medulla. | Multi-site | — |
| BJ050 | Acute infarction involving the right cerebellum, and right pons. | Multi-site | — |
| BJ051 | Acute lacunar infarction involving the left cerebellar hemisphere, and pons. | Multi-site | Lacunar infarction |
| BJ052 | Acute infarction involving the left cerebellum. | Cerebellum | — |
| BJ053 | Acute infarction involving the right pons. | Brainstem | — |
| BJ054 | Acute large infarction involving the right cerebellum. | Cerebellum | Large lesion |
| BJ055 | Acute infarction involving the left cerebellar hemisphere, and pons. | Multi-site | — |
| BJ056 | Acute infarction involving the right cerebellum. | Cerebellum | — |
| BJ057 | Acute infarction involving the left cerebellum. | Cerebellum | — |
| BJ058 | Acute infarction involving the left cerebellum. | Cerebellum | — |
| BJ059 | Acute infarction involving the right cerebellum, right pons, and midbrain. | Multi-site | — |
| BJ060 | Fresh focal infarction involving the right cerebellar hemisphere. | Cerebellum | Fresh infarct wording |
| BJ061 | Fresh infarction involving the left cerebellar hemisphere, and medulla. | Multi-site | Fresh infarct wording |
| BJ062 | Acute infarction involving the left cerebellum. | Cerebellum | — |
| BJ063 | Acute infarction involving the left cerebellum, and left medulla. | Multi-site | — |
| BJ064 | Acute infarction involving the right cerebellum. | Cerebellum | — |
| BJ065 | Acute lacunar infarction involving the left cerebellar hemisphere. | Cerebellum | Lacunar infarction |
| BJ066 | Acute infarction involving the left cerebellum, and left pons. | Multi-site | — |
| BJ067 | Acute multiple infarction involving the cerebellar tonsil. | Cerebellum | Multiple lesions |
| BJ068 | large infarction involving the right cerebellum, medulla, pons, and midbrain. | Multi-site | Large lesion |
| BJ069 | Acute infarction involving the left cerebellar hemisphere. | Cerebellum | — |
| BJ070 | Acute infarction involving the right cerebellum. | Cerebellum | — |
| BJ071 | Acute infarction involving the left cerebellum. | Cerebellum | — |
| BJ072 | Acute infarction involving the left cerebellum. | Cerebellum | — |
| BJ073 | Acute infarction involving the right cerebellum. | Cerebellum | — |
| BJ074 | Acute infarction involving the left cerebellum, and pons. | Multi-site | — |
| BJ075 | Acute infarction involving the right cerebellum. | Cerebellum | — |

Summary (descriptive only; n = 143): Anterior-circulation wording in original MRI-DWI reports (n = 1; not used as the primary APCI site category), Brainstem (n = 21), Cerebellum (n = 67), Multi-site (n = 32), Occipital lobe (n = 1), Thalamus (n = 21).

Note: This table presents available case-level MRI-DWI report wording for 143 of the 161 participants from the two participating hospitals. De-identified study codes were used; BJ001-BJ075 denote records from Baoji High-tech Hospital. The complete-cohort descriptive classification of primary infarct site is reported in Table 4. Supplementary Table S1 is provided for transparency and should not be interpreted as a complete lesion-classification table. Patients with clinically relevant anterior-circulation infarction as the index event were excluded according to the eligibility criteria.

**Supplementary Table S2. Exploratory distribution of NIHSS-defined stroke severity by documented lesion-location category in a one-center subset (n = 75)**

| **Documented lesion-location category** | **Mild stroke, n (%)** | **Moderate-to-severe stroke, n (%)** | **Total, n** |
| --- | --- | --- | --- |
| Cerebellum | 47 (97.9) | 1 (2.1) | 48 |
| Brainstem | 2 (66.7) | 1 (33.3) | 3 |
| Occipital lobe | 1 (100.0) | 0 (0.0) | 1 |
| Multi-site | 7 (30.4) | 16 (69.6) | 23 |
| Total | 57 (76.0) | 18 (24.0) | 75 |

Note: Values are presented as n (%), with percentages calculated within each lesion-location category. Mild stroke was defined as NIHSS < 8, and moderate-to-severe stroke was defined as NIHSS ≥ 8. Lesion-location categories were assigned according to the available MRI-DWI report wording. The distribution of NIHSS-defined stroke severity differed across lesion-location categories (Fisher–Freeman–Halton exact P < 0.001). This exploratory analysis included only 75 participants from one participating center with linkable case-level NIHSS scores and lesion-location descriptions and should not be interpreted as a complete cohort-level territorial analysis. NIHSS, National Institutes of Health Stroke Scale; MRI-DWI, magnetic resonance imaging diffusion-weighted imaging.
